# Job stress and loneliness among desk workers during the COVID-19 pandemic in Japan: focus on remote working

**DOI:** 10.1265/ehpm.22-00107

**Published:** 2022-08-11

**Authors:** Fuyu Miyake, Chimed-Ochir Odgerel, Ayako Hino, Kazunori Ikegami, Tomohisa Nagata, Seiichiro Tateishi, Mayumi Tsuji, Shinya Matsuda, Tomohiro Ishimaru

**Affiliations:** 1 Department of Environmental Epidemiology, Institute of Industrial Ecological Sciences, University of Occupational and Environmental Health, Japan, Kitakyushu, Japan; 2 Department of Public Health and Health Policy, Hiroshima University, Hiroshima, Japan; 3 Department of Mental Health, Institute of Industrial Ecological Sciences, University of Occupational and Environmental Health, Japan, Kitakyushu, Japan; 4 Department of Work Systems and Health, Institute of Industrial Ecological Sciences, University of Occupational and Environmental Health, Japan, Kitakyushu, Japan; 5 Department of Occupational Health Practice and Management, Institute of Industrial Ecological Sciences, University of Occupational and Environmental Health, Japan, Kitakyushu, Japan; 6 Disaster Occupational Health Center, Institute of Industrial Ecological Sciences, University of Occupational and Environmental Health, Japan, Kitakyushu, Japan; 7 Department of Environmental Health, School of Medicine, University of Occupational and Environmental Health, Japan, Kitakyushu, Japan; 8 Department of Preventive Medicine and Community Health, School of Medicine, University of Occupational and Environmental Health, Japan, Kitakyushu, Japan

**Keywords:** Loneliness, Mental health, Occupational stress, Remote work, Telecommuting

## Abstract

**Background:**

Previous studies have reported an increase in loneliness since the outbreak of coronavirus disease 2019 (COVID-19), but there are few data on the relationship between job stress and loneliness. This study aimed to assess the relationship between job stress and loneliness among desk workers, with a focus on the impact of remote working.

**Methods:**

This study was part of the Collaborative Online Research on the Novel-coronavirus and Work (CORoNaWork) project in Japan. We extracted data from 13,468 workers who indicated that they were doing desk work. Loneliness was assessed using a single question and job stress was valuated using the Job Content Questionnaire (JCQ). Multiple logistic regression was performed.

**Results:**

Participants who worked remotely 4 or more days per week were marginally more likely to report feeling lonely compared with those who did not work remotely (adjusted odds ratio = 1.23, 95% CI: 0.99–5.84, *P* = 0.066). Remote working did not explain the interaction between JCQ scale scores and loneliness. Among remote workers, the level of support provided by co-workers and supervisors was strongly associated with feelings of loneliness as well as non-remote workers (co-worker support: AOR = 4.06, 95% CI: 2.82–5.84, *P* < 0.001; supervisor support: AOR = 2.49, 95% CI: 1.79–3.47, *P* < 0.001).

**Conclusions:**

To reduce loneliness and the risk of associated mental health problems, high-frequency remote workers should interact with supervisors and co-workers using the information and communication technology developed for this purpose.

## Background

Loneliness, which has recently become a global concern, is generally defined as a discrepancy between an individual’s preferred and actual levels of interaction in society [1]. This discrepancy is related to anxiety and distress because of the negative experience of feeling alone [2]. Loneliness can increase an individual’s risk of death by over 20% [3], and it has been associated with an increased risk of stroke and coronary-artery disease [4]. In addition to physical effects, loneliness is related to mental health issues. It is a risk factor for future depression, and loneliness with severe depression is related to early death [5]. Therefore, loneliness is associated not only with stressful and unpleasant feelings but also with critical physical and psychological health issues. Although loneliness is often considered to be an issue affecting older people, it is also a risk for younger people [6]. According to a recent study on loneliness in adults [7], the prevalence of loneliness among those aged 19–65 was around 40% to 48%, showing that loneliness is a critical issue for the working generation. Although many variables are related to loneliness among adults, the major factors are considered to be socioeconomic status and income [8]. In addition, high population density is robustly correlated with loneliness [9]. Living alone and the frequency of communication with neighbours have also been shown to be associated with loneliness [10]. With the development of the coronavirus disease 2019 (COVID-19) pandemic, concerns about the impact of loneliness and related factors have amplified.

Since the COVID-19 pandemic began, work styles have changed dramatically, especially for desk workers. The Japanese government established a policy in February 2020 to prevent the spread of COVID-19. In this policy, the implementation of telework by companies was highly recommended [10]. Given the continued spread of the disease throughout Japan, a national state of emergency was declared in April 2020. This order placed a greater emphasis on telework and staying at home [11]. As a result, in Japan, the percentage of companies implementing telework climbed from 26% in March 2020 to 67% May 2020 [12]. Even after the state of emergency ended, companies continued to implement anti-COVID-19 measures by combining in-person work with remote work [13]. Despite the extensive nature of these changes, the impact of job stress on remote workers is unknown, especially in terms of loneliness. Previous research on teleworking in other countries has indicated that remote workers find it difficult to establish social relationships with other workers and that telework can induce feelings of loneliness [14]. Accordingly, office workers have been encouraged to spend at least one-fifth of their work time in the office to prevent isolation [15].

Various issues related to job stress in the workplace have arisen with the rapid establishment of telework during the COVID-19 pandemic. Karasek’s Job Demand-Control-Support model is a leading theoretical model for describing job stress [16]. The theory describes how job characteristics affect the psychological well-being of employees. Decision latitude with respect to work, psychological demands, support from supervisors, and support from co-workers are four key factors that determine job characteristics. Decision latitude is the potential control that a working individual has in making work-related decisions. Previous research shows that lower support, lower decision latitude, and higher psychological job demands can lead to stress and health issues [17, 18]. In addition, remote workers have complex relationships with other workers, and some workers tend to perceive support from co-workers as lower when they are remote versus working at the office [19].

Loneliness was a critical societal issue before the COVID-19 pandemic. When physical distancing was introduced as a societal strategy to prevent the spread of COVID-19, loneliness increased. Although the frequency of remote work has rapidly increased as a result of COVID-19, the relationship between job stress and loneliness has not been fully evaluated. Several studies have revealed an increase in loneliness since the outbreak of COVID-19 [20], but no reports have clarified how job stress influences loneliness among remote workers during the pandemic. Therefore, the objective of this study was to assess the relationship between job stress and loneliness among desk workers, with a focus on remote working. The results will be useful for developing interventions to improve the work environment for remote workers experiencing loneliness.

## Methods

**Fig. 1:**
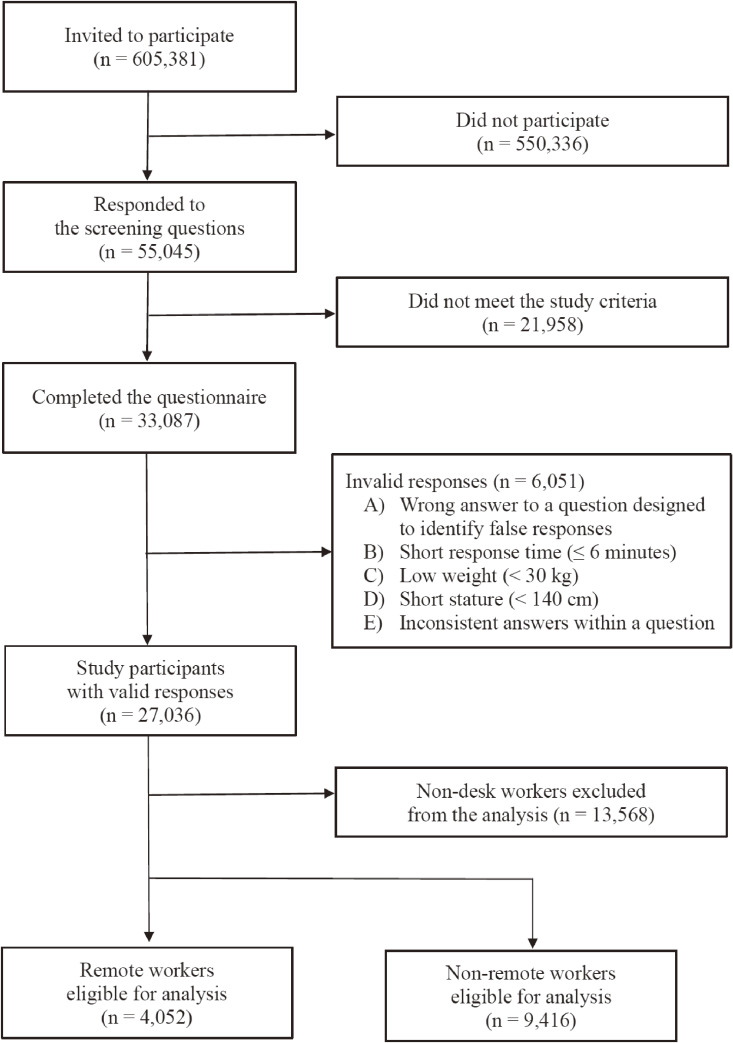
Flow chart of eligible participants in the current study

We performed a cross-sectional survey on the effect of COVID-19 among the working-age population in Japan on December 22–26, 2020, as a component of the Collaborative Online Research on the Novel-coronavirus and Work (CORoNaWork) project [21]. Briefly, the CORoNaWork Project is an anonymous web-based cross-country survey administered during the third wave of COVID-19 infections in Japan. The study targeted individuals registered with Cross Marketing Inc. (Tokyo, Japan). Of these individuals, 605,381 were selected by random sampling and sent an e-mail inviting them to participate in the study. As a result, 55,045 individuals responded to the screening questions and participated in the study. Of those, 33,302 individuals met the survey criteria (age, sex, region, and worker status). We conducted cluster sampling to stratify the respondents by sex, occupation, and region according to COVID-19 incidence rates. As a result, a total of 33,087 individuals completed the questionnaire. After excluding invalid answers, data from 27,036 participants were included in the analysis. In the current study, from these 27,036 participants, we selected 13,468 individuals who indicated in their survey responses that they were desk workers (Fig. 1). The study was approved by the Ethics Committee of the University of Occupational and Environmental Health, Japan (Approval number: R2-079).

The original questionnaire used in the CORoNaWork project consists of 54 main questions, including items on general demographic characteristics, socioeconomic characteristics, work-related characteristics, lifestyle factors, quality of life, health conditions, and COVID-19-related issues (e.g., preventive measures taken by individuals and at the workplace, vaccination, telework, and lifestyle changes during COVID-19). For the current study, we included questions on demographic characteristics (age and sex), socioeconomic characteristics (education, annual household income, and household composition), regional state-of-emergency status, frequency of remote work, job stress, and loneliness.

We asked the participants whether they felt lonely during the study period. Loneliness was evaluated by a single question: ‘Do you feel alone?’ The answer options were *yes* and *no*. This question is included in the Japanese version of the University of California, Los Angeles (UCLA) Loneliness Scale [22].

We included the frequency of remote work and job stress as independent variables in this study. The frequency of remote work was categorized as never, once per week, 2 or 3 days per week, or 4 or more days per week. We used the Job Content Questionnaire (JCQ) to assess job stress in remote workers. The JCQ is a self-administered tool that was proposed by Karasek in 1985 and was invented to assess social and psychological stressors at work based on theoretical models [17]. The original instrument comprises 45 core items; however, in 1995, Japanese researchers first translated the JCQ and developed the Japanese version of the JCQ, which consists of 22 items. The JCQ covers a variety of job characteristics: psychological job demands, decision latitude, support from supervisors, and support from co-workers. The reliability and validity of the questionnaire were verified among the employees of infrastructure companies in the Chubu region [18] and among the workers at a computer company [23]. The average score and reliability coefficient of the Japanese version of the JCQ are very similar to the results in other countries; thus, the JCQ is considered to be internationally applicable in occupational settings [17]. We used the Japanese version of the JCQ to evaluate job stress in the current study.

First, we generated descriptive statistics for the demographic and socioeconomic characteristics of the participants according to whether they were remote or non-remote workers. Each of the 22 JCQ items on the JCQ is answered using a 4-point scale ranging from 1 (*strongly disagree*) to 4 (*strongly agree*). For the assessment, the weighted item scores were summed to produce scores on the following four scales, following the authors of the Japanese version of the JCQ: the psychological demands scale (five items, range: 12–48), the decision latitude scale (nine items, range: 24–96), the co-worker support scale (four items, range: 4–16), and the supervisor support scale (four items, range: 4–16) [18]. The 22 JCQ items include a 5-item psychological demand scale, a 9-item decision latitude scale, a 4-item co-worker support scale, and a 4-item supervisor support scale. For each scale, the relevant items were weighed and calculated according to the impact on the scale. On the basis of the sample distribution in remote workers, each sub-scale was classified into tertiles. According to previous research [17, 18], we decided to use the high scoring group as a reference for high co-worker support, supervisor support, and decision latitude, and the low scoring group as a reference for high psychological job demands for our analysis.

Second, we performed logistic regression analysis to identify the association between the frequency of remote work and loneliness for all participants. Third, we assessed interactions between work status (remote or non-remote) and scores on the four JCQ scale and loneliness, respectively. Finally, we stratified the participant group according to whether they were remote or non-remote workers and then conducted logistic regression analysis to evaluate the associations between the frequency of remote work (remote working group only) and the four JCQ scale scores with respect to loneliness, respectively. We show the results of both the univariate model and the model adjusting for sex, age, education, income, household composition, and regional state-of-emergency status. Statistical significance was defined as *P* < 0.05. Stata/SE 16.1 (StataCorp, College Station, TX, USA) was used for statistical analyses.

## Results

Data from a total of 13,468 desk workers were analysed in the current study. Table 1 indicates the general characteristics of the participants who engaged in remote working (n = 4,052) and non-remote working (n = 9,416). Around half of the respondents were male, were aged 50–65 years, and were university/graduated educated. Of the remote workers participating in the study, 2,042 (50.4%) worked remotely 4 or more days per week, 1,058 (26.1%) worked remotely 2 or 3 days per week, and 952 (23.5%) worked remotely 1 day per week. Regarding job stress, almost half of all remote workers felt a high level of support from their co-workers (46.3%) and supervisors (48.5%). A total of 191 (4.7%) remote workers and 449 (4.8%) non-remote workers reported feeling lonely.

**Table 1:** General characteristics of the study participants

	**Remote working**		**Non-remote working**	
	
	**Total** **N = 4,052**	**Loneliness** **n = 191**	**Total** **N = 9,416**	**Loneliness** **n = 449**
	
	**n (%)**	**n (%)**	**n (%)**	**n (%)**
Male	2,363 (58.3)	102 (53.4)	4,533 (48.1)	161 (35.9)
Age (years)				
20–39	807 (20.0)	57 (29.8)	2,171 (23.0)	151 (33.6)
40–49	1,128 (27.8)	59 (30.9)	2,952 (31.4)	161 (35.9)
50–65	2,117 (52.2)	75 (39.3)	4,293 (45.6)	137 (30.5)
Education				
Junior high or high school	617 (15.2)	39 (20.4)	2,401 (25.5)	123 (27.4)
Vocational school or college	734 (18.1)	41 (21.5)	2,046 (21.7)	105 (23.4)
University or graduate school	2,701 (66.7)	111 (58.1)	4,969 (52.8)	221 (49.2)
Annual household income (Japanese yen)				
< 4 million	890 (22.0)	83 (43.4)	2,157 (22.9)	169 (37.6)
≥ 4 million and < 8 million	1,554 (38.4)	63 (33.0)	4,212 (44.7)	182 (40.5)
≥ 8 million	1,608 (39.6)	45 (23.6)	3,047 (32.4)	98 (21.9)
Household composition				
Single	912 (22.5)	71 (37.2)	1,822 (19.4)	163 (36.3)
Couple	1,156 (28.5)	50 (26.2)	2,552 (27.1)	93 (20.7)
3 or more persons	1,984 (49.0)	70 (36.6)	5,042 (53.5)	193 (43.0)
Regional state-of-emergency status (13 prefectures)*	2,567 (63.4)	113 (59.2)	3,381 (35.9)	149 (33.2)
Co-worker support				
High (12–16 points)	1,877 (46.3)	46 (24.1)	3,976 (42.2)	101 (22.5)
Moderate (10 or 11 points)	1,170 (28.9)	38 (19.9)	3,294 (35.0)	135 (30.1)
Low (4–9 points)	1,005 (24.8)	107 (56.0)	2,146 (22.8)	213 (47.4)
Supervisor support				
High (12–16 points)	1,965 (48.5)	57 (29.8)	4,201 (44.6)	106 (23.6)
Moderate (9–11 points)	609 (15.0)	18 (9.5)	1,653 (17.6)	75 (16.7)
Low (4–8 points)	1,478 (36.5)	116 (60.7)	3,562 (37.8)	268 (59.7)
Psychological job demand				
High (32–48 points)	1,362 (33.6)	66 (34.6)	1,796 (19.1)	70 (15.6)
Moderate (27–31 points)	1,341 (33.1)	53 (27.7)	2,998 (31.8)	101 (22.5)
Low (12–26 points)	1,349 (33.3)	72 (37.7)	4,622 (49.1)	278 (61.9)
Decision latitude				
High (71–96 points)	1,262 (31.1)	49 (25.7)	2,711 (28.8)	121 (26.9)
Moderate (63–70 points)	1,628 (40.2)	67 (35.1)	3,615 (38.4)	136 (30.3)
Low (26–62 points)	1,162 (28.7)	75 (39.2)	3,090 (32.8)	192 (42.8)
Frequency of remote work				
1 day/week	952 (23.5)	28 (14.7)		
2 or 3 days/week	1,058 (26.1)	49 (25.7)		
4 or more days/week	2,042 (50.4)	114 (59.6)		

Regional state of emergency* refers to a government announcement that requires people to refrain from going outside.

Table 2 shows the association between the frequency of remote working and loneliness among desk workers. Initially, we found no significant difference in loneliness between remote and non-remote workers. When remote work was categorized by frequency of remote working, those who worked remotely once a week were less likely to feel loneliness compared with those who did not work remotely. However, this did not reach significance in the adjusted model. Working remotely 4 or more days per week was marginally associated with feeling loneliness (adjusted odds ratio [AOR] = 1.23, 95% confidence interval [CI]: 0.99–5.84, *P* = 0.066).

**Table 2:** Association between the frequency of remote working and loneliness among desk workers (n = 13,468)

	**Univariate**			Adjusted*		
	
	**OR**	**(95% CI)**	***P* value**	**OR**	**(95% CI)**	***P* value**
Remote working						
No	1.00	-	-	1.00	-	-
Yes	0.99	(0.83–1.12)	0.891	1.10	(0.91–1.32)	0.332
1 day/week	0.61	(0.41–0.89)	0.010	0.72	(0.49–1.07)	0.104
2–3 days/week	0.97	(0.72–1.31)	0.843	1.15	(0.84–1.57)	0.393
4 days/week or more	1.18	(0.96–1.46)	0.123	1.23	(0.99–1.53)	0.066

OR: odds ratio; CI: confidence interval

*Adjusted for sex, age, education, annual household income, household composition, and regional state-of-emergency status.

Table 3 shows the association between job stress and loneliness according to whether workers were remote or non-remote. Among remote workers, participants who worked remotely 4 or more days per week had significantly greater odds of feeling lonely than those who worked at home once per week (AOR = 1.60, 95% CI: 1.04–2.46, *P* = 0.033). Participants who reported having a low level of co-worker support had greater odds of feeling lonely than those who were highly supported by their co-workers (AOR = 4.06, 95% CI: 2.82–5.84, *P* < 0.001). Those who were less supported by their supervisors also had greater odds of feeling lonely than those who were highly supported by their supervisors (AOR = 2.49, 95% CI: 1.79–3.47, *P* < 0.001). Compared with those who had low psychological job demands, participants with high demands felt more loneliness (AOR = 2.04, 95% CI: 1.39–2.99, *P* < 0.001). A similar trend was observed among non-remote workers. Remote working did not explain the interaction between JCQ scale scores and loneliness.

**Table 3:** Association between job stress and loneliness in remote and non remote workers

	**Remote workers**						**Non-remote workers**					
	
	**Univariate**			Adjusted*			**Univariate**			Adjusted*		
			
	**OR**	**(95% CI)**	***P* value**	**OR**	**(95% CI)**	***P* value**	**OR**	**(95% CI)**	***P* value**	**OR**	**(95% CI)**	***P* value**
Frequency of remote work												
1 day/week	1.00	-	-	1.00	-	-						
2 or 3 days/week	1.60	(0.99–2.57)	0.049	1.59	(0.98–2.56)	0.059						
4 or more days/week	1.95	(1.28–2.97)	0.002	1.60	(1.04–2.46)	0.033						
Co-worker support												
High (12–16 points)	1.00	-	-	1.00	-	-	1.00	-	-	1.00	-	-
Moderate (10 or 11 points)	1.34	(0.86–2.07)	0.191	1.33	(0.85–2.06)	0.209	1.64	(1.26–2.13)	<0.001	1.60	(1.23–2.08)	0.001
Low (4–9 points)	4.74	(3.33–6.76)	<0.001	4.06	(2.82–5.84)	<0.001	4.23	(3.32–5.39)	<0.001	3.80	(2.97–4.82)	<0.001
Supervisor support												
High (12–16 points)	1.00	-	-	1.00	-	-	1.00	-	-	1.00	-	-
Moderate (9–11 points)	1.02	(0.60–1.75)	0.944	1.05	(0.61–1.80)	0.872	2.85	(2.06–3.94)	<0.001	1.73	(1.28–2.35)	<0.001
Low (4–8 points)	2.85	(2.06–3.94)	<0.001	2.49	(1.79–3.47)	<0.001	3.14	(2.50–3.95)	<0.001	2.85	(2.26–3.60)	<0.001
Psychological job demand												
High (32–48 points)	1.71	(1.18–2.47)	0.004	2.04	(1.39–2.99)	<0.001	1.42	(1.12–1.79)	0.003	1.43	(1.12–1.81)	0.003
Moderate (27–31 points)	1.06	(0.73–1.55)	0.752	1.10	(0.75–1.62)	0.619	0.84	(0.65–1.08)	0.162	0.85	(0.66–1.10)	0.223
Low (12–26 points)	1.00	-	-	1.00	-	-	1.00	-	-	1.00	-	-
Decision latitude												
High (71–96 points)	1.00	-	-	1.00	-	-	1.00	-	-	1.00	-	-
Moderate (63–70 points)	0.81	(0.56–1.17)	0.258	0.80	(0.55–1.17)	0.250	0.86	(0.63–1.17)	0.339	0.78	(0.57–1.06)	0.777
Low (26–62 points)	1.11	(0.79–1.56)	0.560	0.99	(0.69–1.42)	0.945	1.58	(1.21–2.06)	0.001	1.26	(0.95–1.66)	0.107

OR: odds ratio; CI: confidence interval

*Adjusted for sex, age, education, annual household income, household composition, and regional state-of-emergency status.

## Discussion

The present study revealed no significant relationship between remote work and loneliness, although high frequency remote workers tended to be lonely. In addition, co-worker/supervisor support and psychological job demands were related to loneliness, whereas decision latitude was not for remote workers as well as non-remote workers. These findings supplement the data obtained by other studies that are part of the CORoNaWork project. Although loneliness was identified as one of the main factors in psychological distress [24], the relationship between job stress and psychological distress has been complicated during COVID-19 [25]. Our group reported two reasons for this: one is loneliness related to isolation from others [26], and the other is a positive psychological effect of telework [27]. Our findings provide insight regarding possible strategies for combating loneliness during the COVID-19 pandemic.

Prior research has revealed that telework is associated with isolation and loneliness [17, 28]. Although the association between telework and loneliness in this study was not a dose-response relationship, our results suggest that high frequency remote workers may experience some loneliness. Generally, remote workers are thought to be separated from their colleagues and from work-related social relationships. Consequently, remote workers tend to have fewer opportunities for work-related social interaction and are also distanced from praise from their supervisors. Being physically distanced from the workplace and from one’s colleagues can lead to feelings of isolation and loneliness. This is regarded as the principal problem with telework [28]. There are several factors that appear to modulate the relationship between the frequency of telework and loneliness. One is preference for telework, which affects mental health more strongly than the frequency of telework [29, 30]. For instance, people who prefer to work from home are less stressed, and stress and loneliness are strongly correlated [24]. Another factor is recent advances in information and communication technology (ICT) such as e-mail and chat tools that have provided remote workers with opportunities for real-time interaction [31], which may keep people socially connected and help to overcome feelings of loneliness [32]. Our study was conducted in December 2020, and we assume that most of the remote worker participants had the necessary ICT to work from home. In this situation, using the ICT that has been developed for this purpose might reduce loneliness in remote workers.

Our analysis showed that the levels of support provided by co-workers and supervisors were strongly associated with feelings of loneliness among remote workers as well as non-remote workers. On the basis of this, we conducted a post hoc analysis to compare loneliness according to co-worker support and supervisor support. The results revealed that participants with low levels of co-worker support only were significantly lonelier than those with low levels of supervisor support only (AOR = 3.20, 95% CI: 1.58–6.48, *P* = 0.001). This indicates that co-worker support contributed more to reducing loneliness than did supervisor support. A previous study indicated that the perceived experience of feeling physically distant from one’s co-workers increased loneliness and was stressful for remote workers [33]. Therefore, support and connection among colleagues is as important for remote workers as it is for non-remote workers.

The present study suggests that, although a moderate level of psychological job demands did not affect the presence of loneliness, a high level of psychological job demands was associated with loneliness among remote workers. The presence of loneliness was also not affected by decision latitude. According to Karasek’s Demand–Control Model, employees with higher psychological job demands and lower decision latitude employees are likely to experience a higher level of strain [16]. However, we found that, in terms of loneliness, only a high level of psychological job demands was associated with loneliness. Therefore, our study indicates that lower decision latitude is not always related to loneliness in both remote workers and non-remote workers. A previous study reported that workers who face higher levels of psychological work demands are more likely to work overtime [34]. As a result, one would expect those in jobs with high psychological demands to decrease the time spent communicating with colleagues and supervisors, which might lead to the experience of loneliness. Regarding decision latitude, it is generally thought that those with lower levels of decision latitude are managed or instructed by their supervisors and senior colleagues. To some extent, receiving instructions from others, as a form of communication, may play a role in preventing these workers from feeling lonely, although a low level of decision latitude is also a known stress factor for workers [16].

To the best of our knowledge, the present study is the first to report an association between job stress and loneliness with a focus on remote working during the COVID-19 pandemic. However, our study had several limitations. First, the present study was an Internet-based survey, and the generalizability of our results is thus unclear. However, to increase the external validity and decrease bias as much as possible, we defined the target population after cluster sampling stratified by sex, job type, and region based on COVID-19 incidence rate data. Second, although there are several measurements of loneliness [5], in the present study, the presence of loneliness was assessed through a single question. Therefore, it is difficult to generalize from this result. However, our approach was chosen in reference to a previous study that used a single item to measure loneliness [5, 35]. Courtin et al. discovered in their scoping review that the items most commonly used to measure loneliness were the UCLA Loneliness Scale or its revised version (23 articles) and single questions (15 articles) [5]. Third, we were unable to assess the causal relationship between remote work and the presence of loneliness because this was a cross-sectional study. There have been concerns regarding the possibility of reverse causality in this relationship because certain workers might not choose to work remotely to avoid loneliness. However, workers were not always able to control the frequency of telecommuting during the COVID-19 pandemic. Therefore, we considered the possibility of reverse causality to be low. Fourth, there were cofounding factors in the relationship between loneliness and social status. For example, among the many participants from single households who answered that their psychological job demands were high, it is possible that they felt lonely because they did not have a family member living with them. In this case, the presence or absence of a family member could be a confounding factor in the relationship between psychological job demand and loneliness. Therefore, we adjusted for possible confounding factors in our analysis.

## Conclusion

We found that support from co-workers and supervisors was strongly associated with loneliness among remote and non-remote workers in Japan. To prevent remote workers from feeling lonely and from developing mental health problems related to loneliness, they should engage in interactions with supervisors and co-workers using the ICT developed for this purpose.

## Abbreviations

COVID-19: Coronavirus disease 2019
ICT: Information and communication technology
JCQ: Job Content Questionnaire
